# Exopolysaccharide (EPS) Synthesis by *Oenococcus oeni*: From Genes to Phenotypes

**DOI:** 10.1371/journal.pone.0098898

**Published:** 2014-06-05

**Authors:** Maria Dimopoulou, Marlène Vuillemin, Hugo Campbell-Sills, Patrick M. Lucas, Patricia Ballestra, Cécile Miot-Sertier, Marion Favier, Joana Coulon, Virginie Moine, Thierry Doco, Maryline Roques, Pascale Williams, Melina Petrel, Etienne Gontier, Claire Moulis, Magali Remaud-Simeon, Marguerite Dols-Lafargue

**Affiliations:** 1 Université de Bordeaux, Institut polytechnique de Bordeaux, ISVV, EA 4577, Unité de recherche Oenologie, INRA USC 1366, Villenave d’Ornon, France; 2 Université de Toulouse, INSA, UPS, INP, INRA, CNRS, LISBP, Toulouse, France; 3 BioLaffort, research subsidiary of the Laffort Group, Bordeaux, France; 4 INRA, UMR1083, Sciences pour l’œnologie, Montpellier, France; 5 Université de Bordeaux, Bordeaux Imaging Center, UMS 3420 CNRS - US4 INSERM, Bordeaux, France; Institut Pasteur de Lille, France

## Abstract

*Oenococcus oeni* is the bacterial species which drives malolactic fermentation in wine. The analysis of 50 genomic sequences of *O. oeni* (14 already available and 36 newly sequenced ones) provided an inventory of the genes potentially involved in exopolysaccharide (EPS) biosynthesis. The loci identified are: two gene clusters named *eps1* and *eps2*, three isolated glycoside-hydrolase genes named *dsrO*, *dsrV* and *levO*, and three isolated glycosyltransferase genes named *gtf*, *it3*, *it4*. The isolated genes were present or absent depending on the strain and the *eps* gene clusters composition diverged from one strain to another. The soluble and capsular EPS production capacity of several strains was examined after growth in different culture media and the EPS structure was determined. Genotype to phenotype correlations showed that several EPS biosynthetic pathways were active and complementary in *O. oeni.* Can be distinguished: (i) a Wzy -dependent synthetic pathway, allowing the production of heteropolysaccharides made of glucose, galactose and rhamnose, mainly in a capsular form, (ii) a glucan synthase pathway (Gtf), involved in β-glucan synthesis in a free and a cell-associated form, giving a ropy phenotype to growth media and (iii) homopolysaccharide synthesis from sucrose (α-glucan or β-fructan) by glycoside-hydrolases of the GH70 and GH68 families. The *eps* gene distribution on the phylogenetic tree was examined. Fifty out of 50 studied genomes possessed several genes dedicated to EPS metabolism. This suggests that these polymers are important for the adaptation of *O. oeni* to its specific ecological niche, wine and possibly contribute to the technological performance of malolactic starters.

## Introduction

*Oenococcus oeni*, formerly *Leuconostoc oenos* is the bacterial species which most frequently drives malolactic fermentation (MLF) in wine. Nowadays, MLF is recommended for most red wines (and sometimes for white ones), especially when they are meant to age [1]–[3]. Quantitatively, the main change observed during MLF is the transformation of malic acid into lactic acid. However, many other metabolic transformations occur during MLF which undoubtedly have a major effect on wine quality. In order to better control MLF, the use of *O. oeni* as a malolactic starter was proposed early [4]. Wines are inoculated with selected *O. oeni* strains at the end of or after alcoholic fermentation. However, *O. oeni* strains strongly differ regarding their respective ability to survive and conduct MLF after inoculation in wine [5]–[6]. Comparative genomic as well as less global studies led to identify genes with potential technological interest [2], [7]–[12]. Among the metabolic equipments which could explain the different tolerance to inoculation in wine, the biosynthesis of exopolysaccharides (EPS) was recently examined through genomic studies [12], in wine [13] or through the functional study of specific glucan-synthase [14]. EPS are extracellular polymers composed of sugar monomers. With the few *O. oeni* strains studied, the soluble EPS yields and the EPS monomer composition vary depending on the strain and/or on the growth medium composition [15]. Actually, *O. oeni* is able to synthesize both homo and heteropolysaccharides, via distinct metabolic pathways [16]. Most of the time, the medium viscosity is unaltered after EPS synthesis, with the exception of ropy strains which produce β-glucan [13]–[14], [16]–[18].

Considering that *O. oeni* genome has a limited size (<1.8 Mb), whole genome sequencing appeared to be the best strategy to rapidly assess the diversity of genes associated with EPS biosynthesis present in the *O. oeni* pangenome. We therefore analyzed the 14 genomic sequences available [12], and 36 new sequenced ones. The 50 strains studied displayed divergent EPS production level and represented different genetic groups in the *O. oeni* species [19]–[20]. Glycosyltransferase, glycoside-hydrolase and sugar nucleotide precursor biosynthetic genes were identified and the gene cluster organisation was investigated. The link between *eps* genes and the observed EPS phenotypes as well as the *eps* gene distribution on the *O. oeni* species phylogenetic tree were examined.

## Materials and Methods

### Strains

The names of the *O. oeni* strains studied and their origin are presented in Table 1. *Lactococcus lactis* IL1403 was also used for developing the method for capsule observation by electronic microscopy.

**Table 1:** List and origin of the strains studied.

Strain name^a^	Collection^b^	Origin/commercial name	Accession number^c^
0205	IOEB	Champagne isolate	AZHH00000000
0501	IOEB	Red wine France	AZIP00000000
0502	IOEB	French isolate	AZKL00000000
0607	IOEB	French isolate	AZKK00000000
0608	IOEB	French isolate	AZKJ00000000
1491	IOEB	Red wine France	AZLG00000000
277	IOEB-S commercial	SB3, Laffort France	AZKD00000000
436a	IOEB-S	Red wine Bordeaux France	AZLS00000000
450	IOEB-S commercial	450 PreAc, Laffort, France	AZLT00000000
8417	IOEB	Ropy red wine, France	AZKH00000000
9304	IOEB	Cider France	AZKI00000000
9517	IOEB	Floc de Gascogne France	AZKG00000000
9803	IOEB	Red wine France	AZKF00000000
9805	IOEB	Red wine France	AZKE00000000
ATCC BAA-1163	ATCC	Red wine, France	AAUV00000000*
B10	IOEB	French isolate	AZJW00000000
B129*	AWRI	DSM 20252/ATCC23279, Red wine France	AJPT00000000*
B16	IOEB commercial	B16, Laffort France	AZKC00000000
B202*	AWRI	Australian isolate	AJTO00000000*
B304*	AWRI	Australian isolate	AJIJ00000000*
B318*	AWRI	NCDO 1884, Australia	ALAD00000000*
B418	AWRI	MCW Lallemand	ALAE00000000*
B419	AWRI	Lalvin EQ54 Lallemand	ALAF00000000*
B422	AWRI	Viniflora CHR35, Chr. Hansen	ALAG00000000*
B429	AWRI	Lalvin VP41 Lallemand	ACSE00000000*
B548	AWRI	BL-01 Lallemand	ALAH00000000*
B553*	AWRI	Elios-1 Lallemand	ALAI00000000*
B568*	AWRI	Australian isolate	ALAJ00000000*
B576*	AWRI	Australian isolate	ALAK00000000*
C23	IOEB	Cider Normandy France	AZJU00000000
C28	IOEB	Cider, Bretagne France	AZLE00000000
C52	IOEB	Cider Normandy France	AZLF00000000
CiNe	IOEB	Starter CHR Hansen	AZJV00000000
L18_3	IOEB	Red wine Lebanon	AZLO00000000
L26_1	IOEB	Lebanon isolate	AZLP00000000
L40_4	IOEB	Red wine Lebanon	AZLQ00000000
L65_2	IOEB	Red wine, Lebanon	AZLR00000000
PSU-1	commercial	Red wine USA	NC_008528*
S11	S	Sparkling white wine France	AZJX00000000
S12	S	White wine France	AZLH00000000
S13	S	Red wine France	AZKB00000000
S14	S	Red wine France	AZLI00000000
S15	S	Red wine France	AZLJ00000000
S161	S, commercial	350 PreAc, Laffort France	AZLN00000000
S19	S	Red wine France	AZLK00000000
S22	S	Sparkling white wine Bourgogne France	AZKA00000000
S23	S	white wine, England	AZLL00000000
S25	S	Red wine France	AZJZ00000000
S28	S commercial	B28 PreAc, Laffort, France	AZJY00000000
VF	Commercial	Starter VF, Martin Vialatte	AZLM00000000

a the * indicates that the strain was not available in our laboratory for phenotypic analysis.

b TCC: American type culture collection; AWRI: Australian wine research institute; IOEB: Institut d’Oenologie de Bordeaux, France; S: Sarco, Biolaffort, France.

c The* indicates that the genome sequence was already available in the databases.

### Genome Screening, *eps* Gene Identification and Nomenclature

Genomic sequences were recovered from databases or produced by GeT-PlaGe Genotoul (Castanet Tolosan France) and Macrogen (Seoul Korea) (unpublished). All 36 new sequences were annotated by RAST (Rapid Annotation using Subsystem Technology, rast.nmpdr.org) and Kaas (KEGG Automatic *Annotation* Server) [21]. These sequences have been deposited at DDBJ/EMBL/GenBank under the accession numbers listed in Table 1. The versions described in this paper for *eps* gene content are versions XXXX01000000.

Multilocus sequence typing (MLST) was performed for all strains according to the procedure described by Bilhère et al. [19] with some modifications. The sequence type (ST) of each strain was constructed from six housekeeping genes: *gyrB*, *g6pd, pgm, dnaE, purK* and *rpoB* whose sequences were obtained by genome analysis in Seed Viewer application of RAST. Sequence treatment was performed by using BioEdit 7.2.3 and the phylogenetic tree was constructed by the neighbor-joining method with a Kimura two-parameter distance model, using MEGA 4 software [22]. Bootstrap values were obtained after 1,000 iterations.

From the 3 genomes sequences publicly available at the beginning of our work (genomes of strains *O. oeni* PSU- 1, ATCC BAA-1163 and AWRI B429), we created a database of 82 protein sequences (Table S1, panel initial database), potentially associated with the EPS metabolism including glycosyltransferases, flippases (wzx) and polymerases (wzy) but also glycoside-hydrolases and protein sequences involved in the synthesis of precursors (sugar nucleotides). The 47 other annotated genome sequences were then analyzed for the presence of orthologs of these 82 proteins (BLASTP). Once an ortholog was identified, the gene genomic environment was examined. In addition, all the genes encoding proteins different from those in the initial database (identity <70%), but displaying significant homology (BLASTP or TBLASTX cutoff level of 1e^−30^), suggesting proteins with related enzymatic activity, were listed and their genomic environment was analyzed. A second analysis was done by searching, among the proteins deduced from the annotated genomes, the conserved motifs of glycoside-hydrolases and glycosyltransferases. Both methods gave the same results, i.e. the same list of *eps* genes and proteins. To assign protein functions, we used the Pfam database (http://pfam.sanger.ac.uk/). Glycosyltransferase genes were also assigned to GT families, based on the CAZy database. Genes were named (Table S1) according to the bacterial polysaccharide gene nomenclature (BPGN) system [23]: this system is applicable to all species; it distinguishes different classes of genes and provides a single name for all genes of a given function. The prefix wo–. was chosen in reference to *Oenococcus*. The genes in cluster *eps1* were named *woa-* and those in *eps2* cluster *wob-*, *woc-*, *wod-* and *woe-*. The A majuscule was used only for the initial transferase.

### Growth Media

*O. oeni* was propagated either in Grape juice medium [15] or in a semi defined (SMD) medium specifically developed for EPS production by *O. oeni*. The SMD medium contained: (base) casamino acids 10 g/L, sodium acetate 3.4 g/L, KH_2_PO_4_ 1 g/L, MgSO_4_, 7 H_2_O 0.1 g/L, MnSO_4_, 4 H_2_O 0.1 g/L, ammonium citrate 2.7 g/L, bactotryptone 5 g/L, malate 3 g/L, yeast nitrogen base 6.7 g/L, adenine, uracil, thymine, guanine 5 mg/L each, and a carbohydrate (either glucose 20 g/L or glucose and sucrose, 10 g/L each). The pH was adjusted to 5.0. The carbohydrate solutions were prepared as 10X solutions and were sterilized 20 min at 121°C, while the base was prepared as a 2X solution and sterilized by filtration (0.2 µm cut off). *L. Lactis* was propagated in MRS medium [15].

### EPS Synthesis and Quantification

After a two-week growth in SMD medium at 25°C without agitation, the soluble EPS concentration was measured. The whole culture medium was centrifuged (8,000×g, 5 min, 4°C), and the pellet was removed. Three volumes of ethanol-HCl 1 N (95-5) were added to the supernatant to precipitate the polysaccharides. The tubes were let to stand for 24 hours at 4°C. Then, they were centrifuged (18,000×g, 5 min, 4°C), and the pellet was washed with ethanol (80%vol), centrifuged again, dried for 20 min at 65°C and dissolved in distilled water. The amount of neutral polysaccharides was determined by the anthrone sulfuric acid method [24], using glucose as the standard. For each sample, the polymer precipitation and assays were done in triplicate.

### Immunoagglutination and Capsule Observation

To visualize the bacterial capsule, 10 µl of cell suspension (one week grape juice or SMD culture broth) were deposited on a microscope slide and mixed with 20% nigrosine aqueous solution and let to dry (5 min). Afterwards, 10 µl of 1% crystal violet solution was added and the slide was examined under Olympus BX51 microscope (×100, under oil immersion). The capsule appeared as a white halo around the cells. The β-glucan layer was not sufficiently compact to be visualized by this method. As a result, agglutination tests were performed using *S. pneumoniae* type 37-specific antiserum, as previously reported [14]. Four microliters of antiserum were spotted on a slide with 20 µl of culture broth and incubated 30 min at 4°C before observation using phase contrast microscopy.

For transmission electron microscopy (TEM), bacteria were fixed for 2 hours in 0.1 M sodium cacodylate buffer (pH 7.2) containing 2% glutaraldehyde, at room temperature. Fixed bacteria were stored at 4°C in the fixative solution. They were rinsed in cacodylate buffer, then in 1% gelatin and postfixed (i) with 1% osmium tetroxide containing 1.5% potassium cyanoferrate and (ii) with 3% uranyl acetate at 4°C. They were gradually dehydrated in ethanol (30% to 100%) and embedded in Epon. Thin sections (70 nm) were collected on 150-mesh cooper grids, before examination with a Hitachi H7650 TEM. Negative staining and TEM observation gave the same results (presence or absence of capsule) for all the strains examined.

### EPS Purification and Structural Analysis

For capsule structure determination, 500 mL of SMD-glucose culture medium was centrifuged and the pellet was washed twice with PBS buffer (NaCl 137 mM, KCl 2.7 mM, Na_2_HPO_4_ 10 mM, pH 7). Then the pellet was washed with 100 ml of ultrapure water and the cell walls were recovered by centrifugation (6000×g, 4°C, 20 min) and freeze dried. The capsular polysaccharides were then recovered by the method described by Gorska et al [25].

In order to analyze the soluble EPS produced in SMD-Glucose or SMD-glucose-sucrose, 500 mL of a two-week culture broth were centrifuged (10 000×*g*, 20 min, 4°C), and the supernatant was dialyzed for 48 h against water (MWCO 3500 Da) and freeze dried.

The molecular weight distribution of an aqueous solution of freeze dried soluble EPS was established by high-performance size-exclusion chromatography (HPSEC) using a system composed of a 234-Gilson sampling injector (Roissy, France) and an LC-10 AS Shimadzu pump (Kyoto, Japan). HPSEC elution was performed on two serial Shodex OHPAK KB-803 and KB-805 columns (0.8×30 cm; Showa Denko, Japan), connected to an ERC-7512 refractometer (Erma, Japan), at a 1 mL/min flow rate in 0.1M LiNO_3_. The apparent molecular weights were calculated from the calibration curve established with a Pullulan calibration kit (Showa Denko, Japan).

Neutral monosaccharides were released after polysaccharides hydrolysis by treatment with 2 M trifluoroacetic acid (120°C, 75 min) [26]. The released monosaccharides were methylated using methyl sulfinyl carbanion and methyl iodide [27], and converted to their corresponding alditol acetates by treatment with NaDH_4_ and then acetylated [28]. The methylated residues were quantified by gas chromatography (GC), using a fused silica DB-225 (210°C) capillary column (30 m ×0.32 mm internal diameter, 0.25 µm film), with hydrogen as the carrier gas, on a Shimadzu GC-2010 *plus* gas chromatograph. The alditol acetates were identified from their retention times, by comparison with standards. Neutral sugars amounts were calculated relative to the internal standard (myo-inositol).

The neutral, acidic and amino sugar composition of the EPS was determined after N-reacetylation after solvolysis with anhydrous MeOH containing 0.5 M HCl (80°C, 16 h), and gas chromatography of the per-O-trimethylsilylated methyl glycoside derivatives (TMS). The TMS derivatives were separated on two DB-1 capillary columns (30 m × 0.25 mm i.d., 0.25 µm film) (temperature program 120 to 200°C, 1.5°C/min), coupled with a single injector inlet, through a two-holed ferrule, with H_2_ as the carrier gas, on a Shimadzu GCMS-QP2010SE gas chromatograph. The outlet of one column was directly connected to a FID (250°C). The second column was connected to a mass detector, via a desactived fused-silica column (0.25 m × 0.11 µm i.d.). Samples were injected in pulsed split mode, with a 20∶1 split ratio. The transfer line to the mass was set at 280°C. Electro Ionization (EI) mass spectra were obtained from *m/z* 50 to 400 every 0.2 s, in total ion-monitoring mode (200°C ion source temperature, a 60 µA filament emission current and a 70 eV ionization voltage).

The EPS produced on SMD-Glucose-sucrose were also analyzed for glycosidic linkage. Five mg of EPS in 0.5 ml dimethylsulfoxide were methylated as described above and then hydrolyzed with 2 M trifluoroacetic acid (120°C, 1.15 h). The released methylated monosaccharides were converted to their corresponding alditol acetates. The partially methylated alditol acetates were analyzed by GC-EI-MS on a Shimadzu GCMS-QP2010SE gas chromatograph using a DB-1 capillary column (30 m × 0.25 mm i.d., 0.25 µm film) and the following temperature program: 135°C for 10 min, and rise to 180°C at 1.2°C/min. The transfer line to the mass was set at 280°C. EI mass spectra were obtained from *m/z* 50 to 400 every 0.2 s, in total ion-monitoring mode (200°C ion source temperature, a 60 µA filament emission current and a 70 eV ionization voltage).

## Results

### *eps* Gene Inventory

#### Global analysis

**Figure 1:**
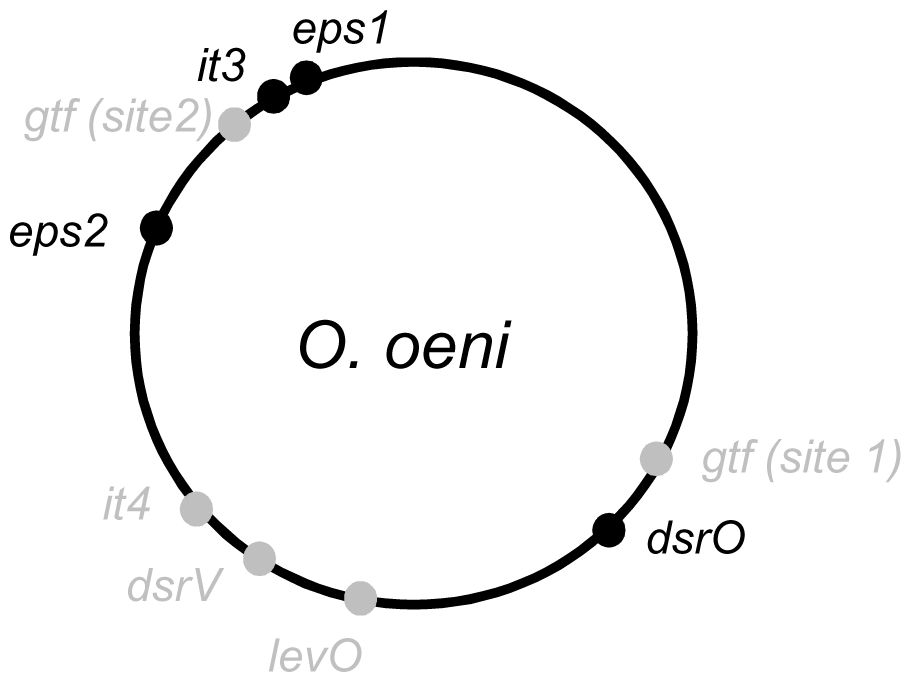
Schematic representation of the *eps loci* on the chromosome of *O. oeni*. The chromosome of *O. oeni* PSU-1 is represented with its own *eps genes or loci* (black). The position of the adjacent regions of the additional *loci* found in other *O. oeni* strains are presented in gray: *eps1* and *eps2*: heteropolysaccharide clusters; *gtf:* β-glucan synthase gene; *it3* and *it4*: priming glycosyltransferase isolated genes; *dsrO* and *dsrV*: dextransucrase genes; *levO*: levansucrase gene.

Many genes potentially associated with EPS biosynthesis were identified: these included glycosyltransferase and glycoside hydrolase genes, either isolated or clustered, and genes associated with the synthesis of nucleotide-sugars or other precursors. These genes are listed in Table S1. Only some of these genes, because (i) their link with EPS metabolism is plausible and (ii) they are not strictly conserved in all the genomes studied, will be presented in detail in this article. All the genes studied were chromosomal (Figure 1). There were two complex heteropolysaccharide clusters, *eps1* and *eps2*, displaying a high density of coding sequences and related to the *eps* clusters previously described by Dimopoulou et al. [16], genes of glycoside-hydrolases (*dsrO, dsrV* and *levO*) and 3 isolated glycosyltransferase genes (*gtf*, *it3* and *it4*). All the genes and clusters studied, when present, were always located at the same site on the bacterial chromosome, except the *gtf* gene which could be found in two different positions in the chromosome (Figure 1). The analysis also indicated that each of the 50 genomes studied was equipped with several distinct genes encoding distinct EPS biosynthetic pathways. This point will be detailed below, *locus* by *locus*.

#### Cluster *eps1*

**Figure 2:**
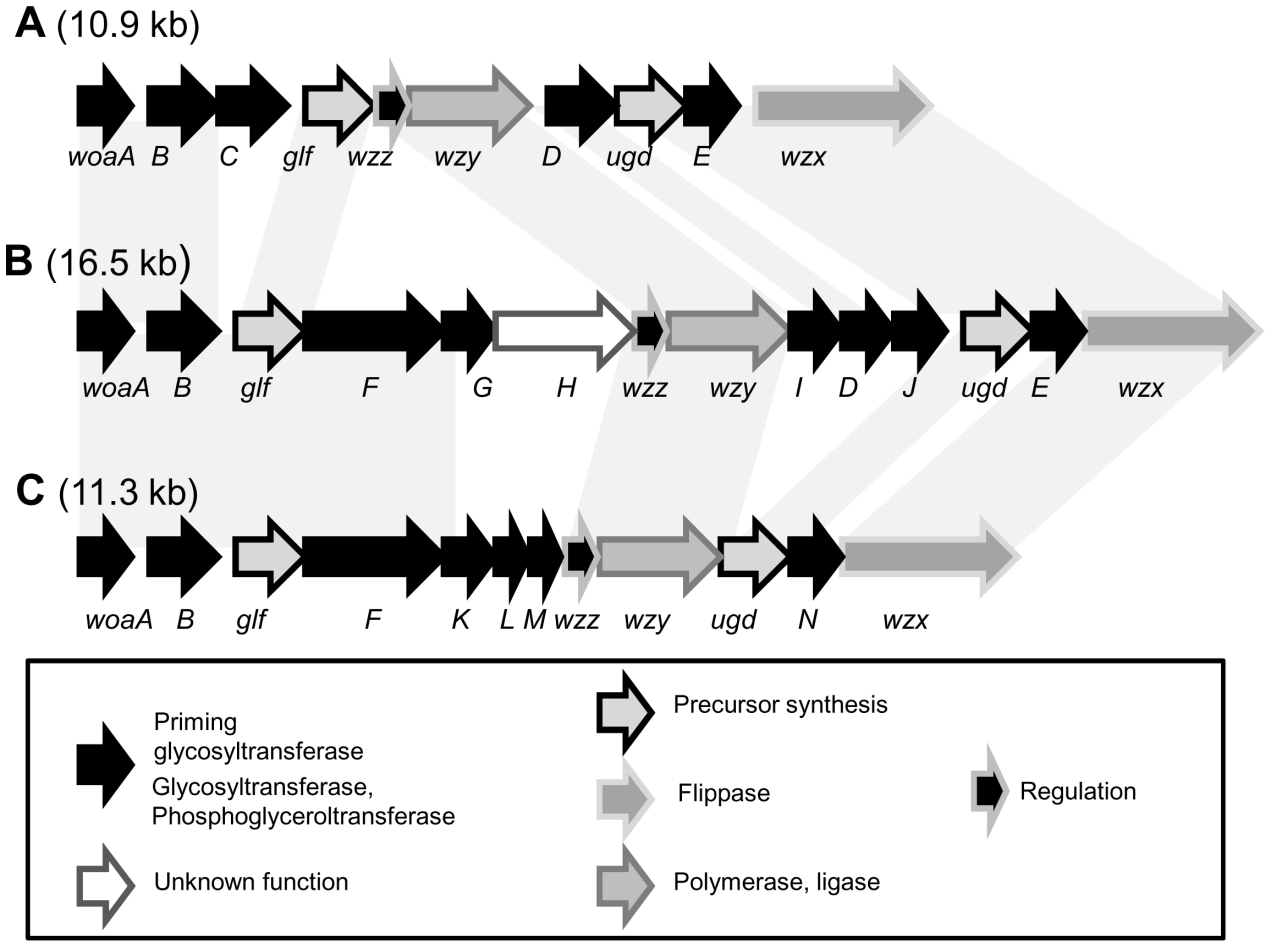
The three models of cluster *eps1*. The arrows filling indicate the putative function of the encoded proteins. The amino acid sequence similarities between the models are shown. **Model A: reference strain** ***O. oeni*** **PSU-1,** other strains: BAA1163, B418, C23, C28, 0501, 0502, 8417, 9304, 9805, 9803, S12, S13, S14. **Model B: reference strain** ***O. oeni*** **B429,** other strains: B202, B304, B318, B553, B568, B576, B10, 1491, L18_3, L40_4, L65_2, 9517, 0608, S161, L26_1, CiNe, 277, 450, S28, 0607, C52, S11, S15, S19, S22, S23, S25. **Model C, reference strain** ***O. oeni*** **B422,** other strains B129, B419, B548, 436a, VF, 0205, B16.

All the genomes studied displayed a *eps1* cluster. The analysis the 50 *eps1* sequences indicated the existence of three related models named A, B and C (Figure 2). Fourteen out of 50 genomes displayed a model A of cluster *eps1*, 28/50 genomes displayed a model B, and the remaining eight genomes had a model C. When two genomes displayed the same model of *eps1*, the cluster gene sequences were over 97% conserved.

The three models of cluster *eps1* differed by the presence of additional genes and by gene synteny. However, more than half of the genes in the cluster were highly conserved (Figure 2, Table 2). The genes encoding UDP-glucose dehydrogenase (*ugd*) and galactopyranomutase (*glf)* were the most conserved ones. The model A was that previously described for strains PSU-1 and BAA-1163 [16]. This was the least complex model of cluster *eps1* regarding the glycosyltransferase gene composition (5 genes, Table 2). Model B differed from model A by the presence of five additional genes (*woaF*, *G*, *H*, *I* and *J*). Model B therefore encoded seven putative glycosyltransferases, a putative phosphoglyceroltransferase WoaF and a protein with unknown function, WoaH. Moreover, WoaD and WoaE were relatively divergent between models B and A (Table 2). In model C, the gene *woaF* was present, as in model B, but genes *woaC*, *D, E, G, H, I* and *J* were absent and new genes were present (*woaK, L M* and *N,* Figure 2). The protein Wzy encoded in model C was highly divergent compared to versions A and B (Table 2).

**Table 2:** Protein sequence identity in *eps1* clusters.

Protein name	Protein size (aa)	GC %	Putative function^a^	Model of *eps1*		
				A^b^	B^b^	C^b^
WoaA	209	40.5	Priming glycosyltransferase	100%	92%	91%
WoaB	250	36.5	Glycosyltransferase NC	100%	92%	87%
Glf	391	36.6	UDP-galactopyranose mutase	100%	99%	97%
Wzz	181	33.0	Polysaccharide synthesis regulation	100%	65%	41%
Wzy	455	29.0	Polymerase or O-antigen ligase	100%	72%	30%
WoaD	312	31.5	Glycosyltransferase NC	100%	67%	39%
Ugd	388	37.8	UDP-glucose 6 dehydrogenase	100%	99%	97%
WoaE	269	26.3	Glycosyltransferase GT-2	100%	68%	Abs
Wzx	479	29.2	flippase	100%	91%	51%
WoaF	652	37.9	glycerophosphotransferase	Abs	100%	65%
Strains displaying the model out of 50				14/50	28/50	8/50

a NC: No Cazy number.

b Identity (%) between proteins of selected strains representative of each model :*O. oeni* PSU-1 (model A) is used as a reference, and ortholog proteins of strain *O. oeni* B429 (model B) and *O. oeni* B422 (model C) are compared to *O. oeni* PSU-1 ones, except for WoaF, for which the sequence found in *O. oeni* B-429 is used as the reference. When two strains display the same model of cluster *eps1*, the identity between related proteins is higher than 98%. Abs: protein absent.

Whatever the model, the cluster apparently brought all the information necessary for the establishment of a heteropolysaccharide biosynthetic pathway: a priming glycosyltransferase gene *woaA*, genes encoding glycosyltransferases potentially associated with the synthesis of the repeating unit (*woaB* to *woaN*) or to precursor synthesis, *glf* and *ugd*. The functional annotation of Ugd, Glf and WoaF suggests the presence of glucuronic acid, phosphoglycerol and galactose in the synthesized product. The *wzz* gene encoded a protein which exhibited little homology in the data bases, but may participate in the regulation of the biosynthetic pathway (chain length regulation). The cluster also comprised a flippase gene, *wzx*, and a potent polymerase gene, *wzy.* Indeed, whatever the model of cluster *eps1* considered, the gene *wzy* was very singular. It may encode a polysaccharide polymerase (Wzy) and, in this case, the cluster encodes a complete heteropolysaccharide biosynthetic pathway. However, the analysis of conserved domains (PFAM hidden Markov models (HMM) Table S1, panel *eps1*) and the analysis of membrane spanning domains (not shown) suggest that it might rather be a O- antigen ligase (Wzy-C superfamily, WaaL,). Enzymes of this family catalyze the binding of polysaccharides moieties of lipopolysaccharide on the oligosaccharide core anchored in the lipid membrane in Gram negative bacteria [29] However, such an activity has never been described in Gram-positive bacteria.

#### Cluster *eps2*

**Figure 3:**
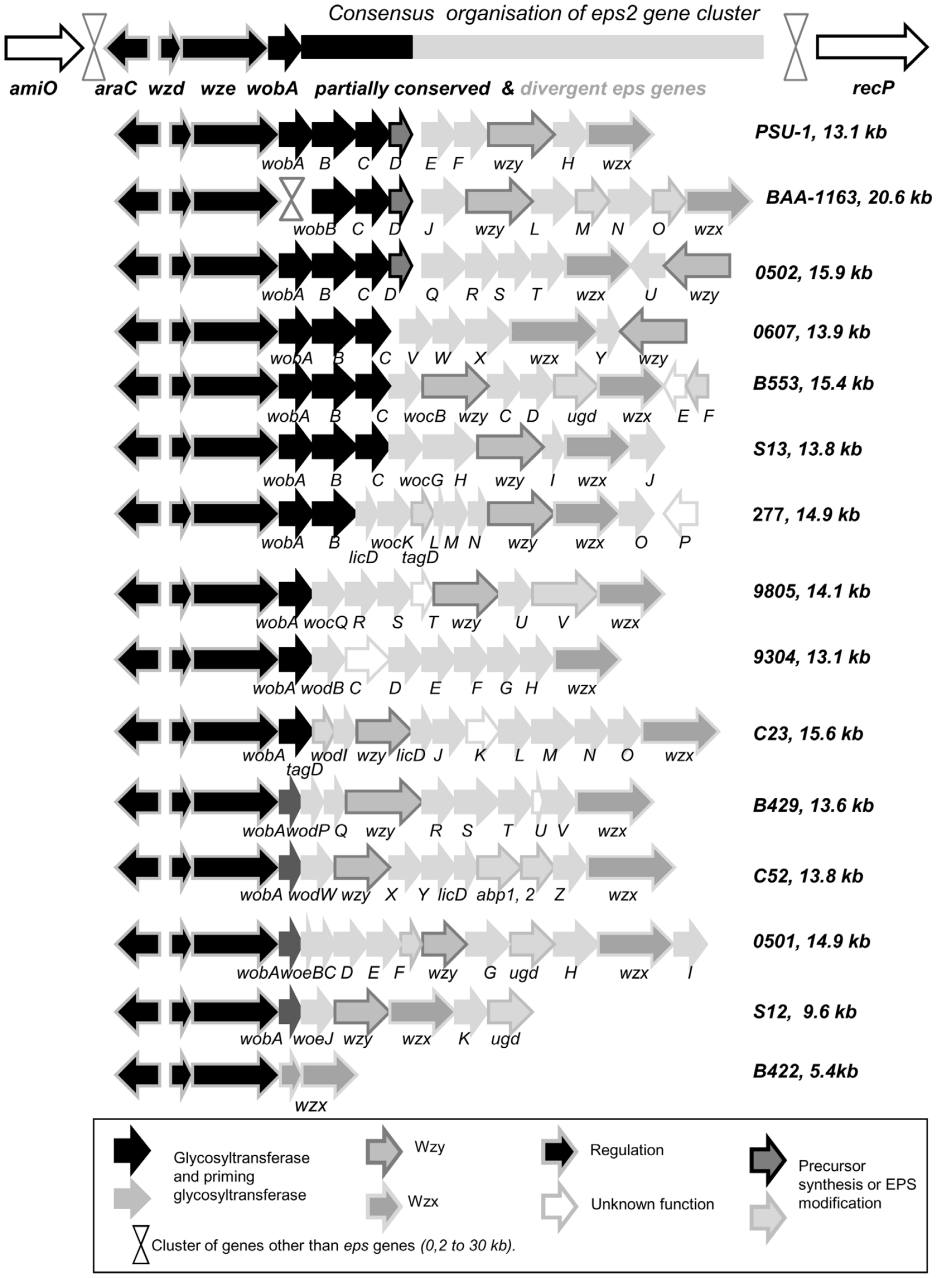
Comparison of the *eps 2* gene clusters. In front of each model of cluster *eps2*, the name of the model strain and the size of the cluster are indicated. When present, the *eps2* cluster is always located between *recP* and *amiO (*core genome genes in *O. oeni* chromosome). It displays, in its 5′ end, the three genes *araC*, *wzd* and *wze*, the initial transferase gene *wobA* (3 different versions), and then, genes specific to each model. The arrows filling indicate the putative function of the encoded proteins. The black and dark gray fillings indicate genes shared by several models of *eps2*. On the other hand, light gray arrows indicate genes specific to a single model. Groups of strains bearing the same *eps2* cluster: ***Model PSU-1:*** *B418,* ***Model 0502:*** *B10,* ***Model 0607:*** *L26_1, S22, S25,* ***Model B553:*** *L65_2, 9517* ***Model 277:*** *S15, S161, L18_3, 450, S14,* ***Model*** ** ***9805*** *∶9803, 8417,* ***Model 9304*** *: C28,* ***Model B429:*** *B202, B304 B318, B568, B576, 0608, CiNe, S11, S23, S28,* ***model B422*** *: B548, 0205, B16.* ***No eps2*** *: VF, S19, 1491, B129, L40_4, 436a, B419.*

Forty-three out of fifty genomes displayed a second heteropolysaccharide cluster *eps2*. Fifteen models of cluster *eps2* were identified (Figure 3, Table S1, *eps2* panel). The cluster size ranged from 5.4 kb to 20.6 kb, but 12 out of 15 models had a size of between 13.1 and 15.9 kb. When two genomes displayed the same model of cluster *eps2*, the nucleotide sequence identity was very high (99 to 100% for each gene in the cluster). Cluster *eps2* was always positioned at the same site in the chromosome of *O. oeni*, between an amidase gene, called *amiO* (OEOE_1519 in *O. oeni* PSU-1) on the 5′ end, and the *recP* gene (OEOE_1480 in *O. oeni* PSU-1) on the 3′ end (Figure 3). Genes other than *eps* genes were systematically inserted between genes *amiO* and *recP*. The nature of the additional genes and the total size of the insert varied from strain to strain. The size of the sequence between genes *amiO* and *recP* ranged from 25 to about 50 kb. This chromosome section did not present mobile elements that could explain its high level of plasticity.

With the exception of *araC* and a few other genes, all the genes in cluster *eps2* were oriented in the same direction as genes *amiO* and *recP* (Figure 3). The *araC, wzd* and *wze* regulatory genes were highly conserved in all the genomes that displayed a cluster *eps2*, with strong sequence conservation. They always appeared in the same order and always at the 5′ end of the *eps* cluster, although the sequence upstream *araC*, between *araC* and *amiO,* was highly variable. In most *eps2* clusters (13 out of 15), the fourth gene was *wobA*. This gene encoded the priming glycosyltransferase that initiates the synthesis of the repeating unit. Three alleles of the priming glycosyltransferase gene (*wobA_PSU1_*, *wobA_B429_*, *wobA_S12_*) were found among the 13 models of cluster *eps2* displaying this gene (Figure 3). The protein WobA_B429_ displayed 39% identity with WobA_S12_ and 65% identity WobA_PSU1_, while forms WobA_PSU1_ and WobA_S12_ shared 38% identity. Nine of the 15 models of clusters *eps2* encoded a priming glycosyltransferase related to WobA_PSU1_ (protein identity >85%), three models encoded a priming glycosyltransferase related to WobA_B429_ and model S12 was the sole to encode the allele WobA_S12_. The gene *wobA* was absent in the genome of strain ATCC BAA-1163, but also in that of strains B422, B548, B16 and 0205. In the last four genomes, the cluster *eps2* was highly truncated: next to the conserved regulatory genes, there was only a truncated gene related to a flippase gene, wzx, strongly resembling the flippase gene of PSU-1 *eps2* model (99% nucleotide identity).

Next to the *wobA* gene, most of the models of *eps2* cluster displayed the genes encoding the glycosyltransferases potentially involved in the repeating unit synthesis. The polymerase and flippase genes but also genes encoding enzymes involved in precursor synthesis or modification complete the cluster. The 5′ end of this part of the cluster (beyond *wobA*) was sometimes conserved between genomes (black arrows), whereas the 3′ end was highly divergent (light gray arrows in Figure 3). Indeed, in that 3′end “gray” zone of cluster *eps2*, no nucleotide identity was found between models taken in pairs, except for a few flippase genes (wzx, see below). However, function homologies (same PFAM) between encoded proteins were common. The proteins deduced from genes in this 3′-end of the *eps2* clusters displayed homologies (35 to 85%) with proteins sequenced from very diverse bacteria: *Lactobacillus rhamnosus, Lb casei, Lb fermentum, Lb amylovorus, Lb paracasei, Lb delbrueckii, Lb plantarum, Lb vaginalis, Streptococcus thermophilus, S. pneumoniae, S. sanguis, S. sanguinis, S. agalactiae, Leuconostoc citreum, Ln. mesenteroides, L. lactis, Pediococcus acidilactici, Enterococcus faecalis, Bifidobacterium bifidum, Bacillus coagulans or Bacteroides dorei*. Few of these species are encountered in wine environment, but very few wine bacteria genomes have been sequenced and published at the time of this study.

Sequence analysis of the protein sequences deduced from the 15 models of cluster *eps2* led to identify (Figure 3, Table S1, panel *eps2*):

- 3 highly conserved regulatory proteins (AraC, Wzd, Wze),
- 13 distinct polymerase (Wzy), displaying low identity with the sequences in the database. WodC encoded in model 9304 of *eps2* may be a 14^th^ polymerase,
- 9 flippases families: B422/PSU1 (99% identity), BAA-1163/9805 (80% identity), 0502/9304/0607/C52/C23 (more than 75% identity), 0501, B429, 9517, S13, 277, S12,
- 3 alleles of priming glycosyltransferases WobA (WobA_PSU1_, WobA_B429_, WobA_S12_),
- 5 putative rhamnosyltransferases WobB, WobF, WobJ, WobS and WobU (1GT-1, 4GT-2),
- 4 putative galactosyltransferases, WocK, WocS, WodQ and WodS (1 GT-1, 2 GT-2, 1 GT-28),
- 3 putative choline phosphotransferases (LicD_277_, LicD_C23_, LicD_C52_),
- 1 putative glucosyltransferase, WobE,
- 53 glycosyltransferases, whose substrate specificity could not be predicted by sequence analysis and, among them, 24 glycosyltransferases classified in GT-2, 19 in GT-1, 2 in GT-4 and 8 not associated with a CAZy family,
- 4 putative acetyltransferases and 2 putative pyruvyltransferases,
- 3 UDP-glucose-dehydrogenase (UgdB_553_, Ugd_0501_, Ugd_S12_), 2 glycerol-3-P-cytidyltransferase (TagD_277_, TagD_C23_), 1 nucleotidyl-transferase (Abp1_C52_) and 1 epimerase (Abp2_C52_),
- and 6 proteins with unknown function (WocE, WocP, WocT, WodC, wodK, wodU).

The substrate specificity prediction for glycosyltransferases and others enzymes encoded in clusters *eps1* and *eps2* suggests that the monomers found in the heteropolysaccharides produced by *O. oeni* may be different from one strain to the other. These heteropolysaccharides may be made of either galactose, rhamnose, glucose and/or glucuronic acid. Furthermore, they may be substituted by acetate, pyruvate, choline and glycerol. Other monomers may also be present, given the high proportion of glycosyltransferases whose protein sequence did not enable to predict their substrate specificity. Nevertheless, the strong similarity between the flippases encoded by different models of cluster *eps2* suggests that the repeating units transported may be of relatively close composition or structure, unless these flippases are sufficiently flexible to transport different oligosaccharide structures.

#### Precursors

**Figure 4:**
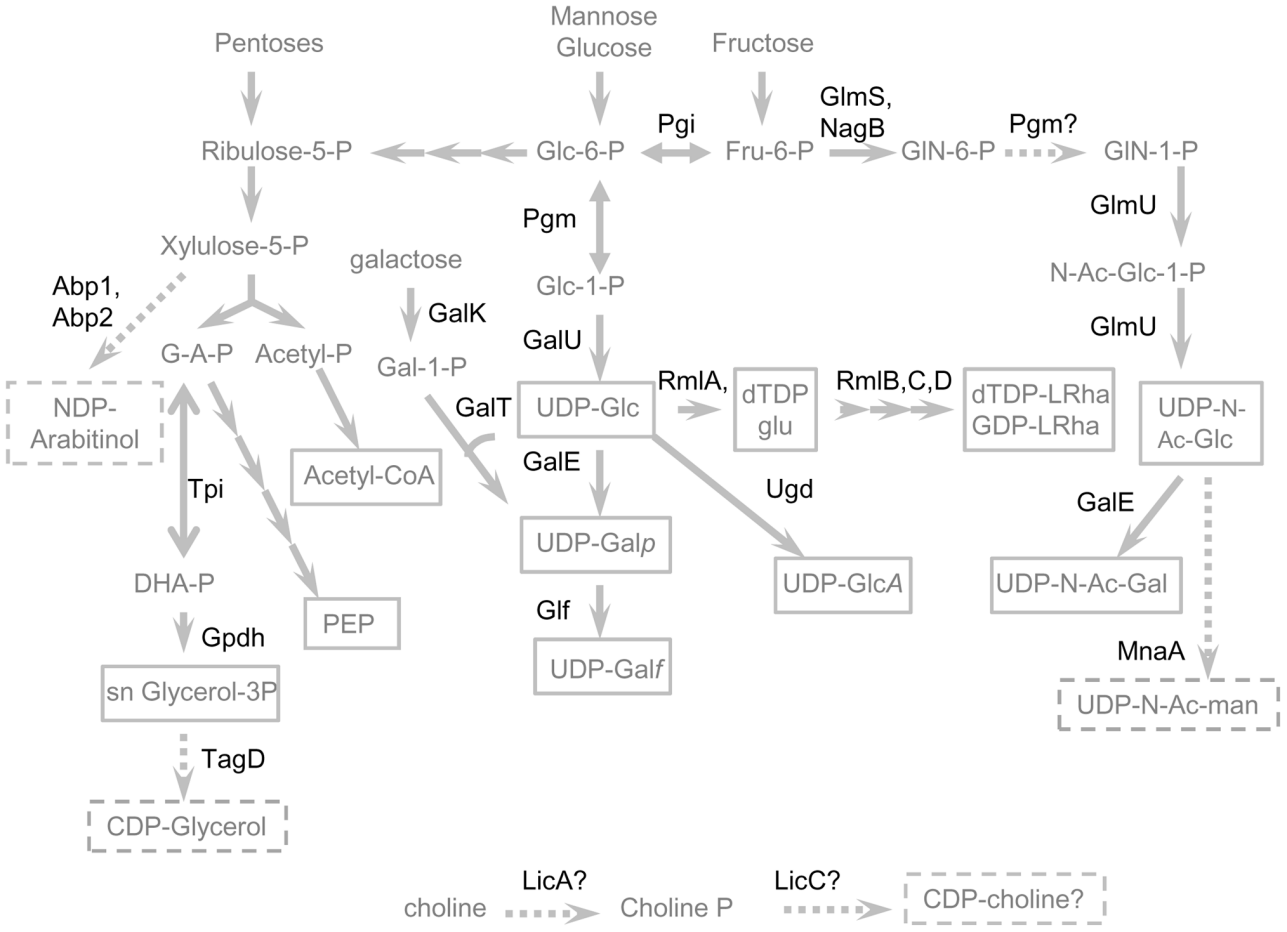
Putative precursor biosynthetic pathways active in *O. oeni* deduced from genome analysis. The enzyme full names and the accession numbers of reference proteins are shown in Table S1 (panel precurors). The solid arrows indicate the central pathways (glucose 6-P to xylulose-5-P and PEP and acetyl-CoA) and the pathways potentially active in all the strains studied, as the associated enzymes are encoded by the 50 genomes studied. The dashed arrows indicate pathways putatively active in a smaller number of strains. The EPS monomer precursors potentially available in all the strains studied are boxed in solid lines, while the precursors putatively available in a limited number of strains are boxed with dotted lines. “?” indicate metabolic steps for which no enzyme was identified from the genome analyses. *P: phosphate, CoA : coenzyme-A, NDP : nucleotidyl-diphosphate, CDP : cytidyl-diphosphate, UDP : uridine-diphosphate; GDP: guanosine-diphosphate, dTDP : desoxythymidine diphosphate, Glc : glucose, Fru : fructose, GlcA : glucuronic acid, Gal : galactose, Galp : galactopyranose, Galf : galactofuranose*, LicA: choline kinase, LicC: choline cytidyltransferase *LRha, L-rhamnose, GlN : glucosamine, N-Ac-Glc : N-acetyl glucosamine, N-Ac-Gal : N-acetyl-galactosamine, N-Ac-Man : N-acetyl-mannosamine, G-A-P : glyceraldehyde 3-phosphate, DHAP: dihydroxyacetone phosphate, PEP : phosphoenolpyruvate.*

Beyond the substrate specificity of the glycosyltransferases in the *eps* clusters, the precursors biosynthetic pathways may also limit the variety of monomers encountered in *O. oeni* heteropolysaccharides [30]–[31]. It is generally accepted that the monomers are transferred from sugar nucleotides (NDP-linked), except for acetyl and pyruvyls which are respectively transferred from acetyl-CoA and phosphoenolpyruvate (PEP). The genes associated with the biosynthesis of these different precursors have been sought in the different genomes (Table S1, panel precursors). Most of these genes were located outside the *eps1* and *2* clusters and formed part of the core genome. Thus, as indicated in Figure 4, all the strains studied were equipped to synthesize PEP, acetyl-CoA, UDP-glucose, UDP-galactopyranose and UDP-galactofuranose, dTDP-rhamnose and dTDP-glucose, UDP-glucuronate and, provided that phosphoglucomutase is able to catalyze the conversion of glucosamine-6-phosphate to glucosamine-1-phosphate, UDP-N -acetylglucosamine and UDP-N-acetylgalactosamine.

On the other hand, only a few strains were apparently able to produce CDP-glycerol (proteinTagD provided by *eps2* models 277 or C23) or UDP-N-Acetyl mannosamine (Mna provided by *eps2* model C52). Regarding the biosynthesis of NDP-arabitinol, the genes *abp1* and *abp2* were found in the C52 genome (in cluster *eps2*) but the deduced proteins exhibited moderate identities with proteins Abp1 and Abp2 found in the databases (37% and 30%). Finally, the biosynthetic pathway for CDP- choline (LicA and LicC) was not found in any of the studied genomes, although three models of cluster *eps2* (8 strains involved) encoded a choline phosphotransferase (LicD). Nevertheless, we cannot exclude that these functions are performed by highly divergent proteins in *O.* *oeni*.

#### Additional glycosyltransferase genes

Another element may contribute to the modulation of the structure of the EPS produced by *O. oeni*: the presence of additional glycosyltransferase genes, outside *eps1* and *eps2* clusters. However, most of the additional glycosyltransferase genes studied formed part of the core genome (Table S1, panel additional glycosyltransferases). It should be noted, among these highly conserved glycosyltransferase genes, the presence of a priming glycosyltransferase gene (*it3*) that could complement truncated *eps* clusters such as the BAA-1163 *eps2* model.

Other genes were present in a smaller number of genomes. Thus, another putative gene of priming glycosyltransferase (*it4*) was present in 8/50 genomes. The analysis of adjacent genes indicated that the acquisition of this gene was probably related to a phage attack (gene in a phage remnant). Furthermore, 5 out of 50 genomes encoded a processive glucosyltransferase, Gtf, 97% identical to the glucosyltransferase described in *Pediococcus parvulus* IOEB 8801, for the biosynthesis of β-1,3 -β-1,2 glucan associated with wine ropiness [17], [32]. The *gtf* gene of *O. oeni* IOEB 0205 was previously characterized [14] but its exact location on the chromosome and its presence in the 4 other genomes were discovered in the present study. Two separate insertion sites were identified for *gtf* (Figure 1). The gene is located within a 15.5 kb insert (phage remnant) in the genome of strains B422, B548, 0205 and B16. In 0502 genome, the *gtf* gene was inserted in a potentially mobile prophage (40.9 kb insert).

#### Glycoside-hydrolases

**Figure 5:**
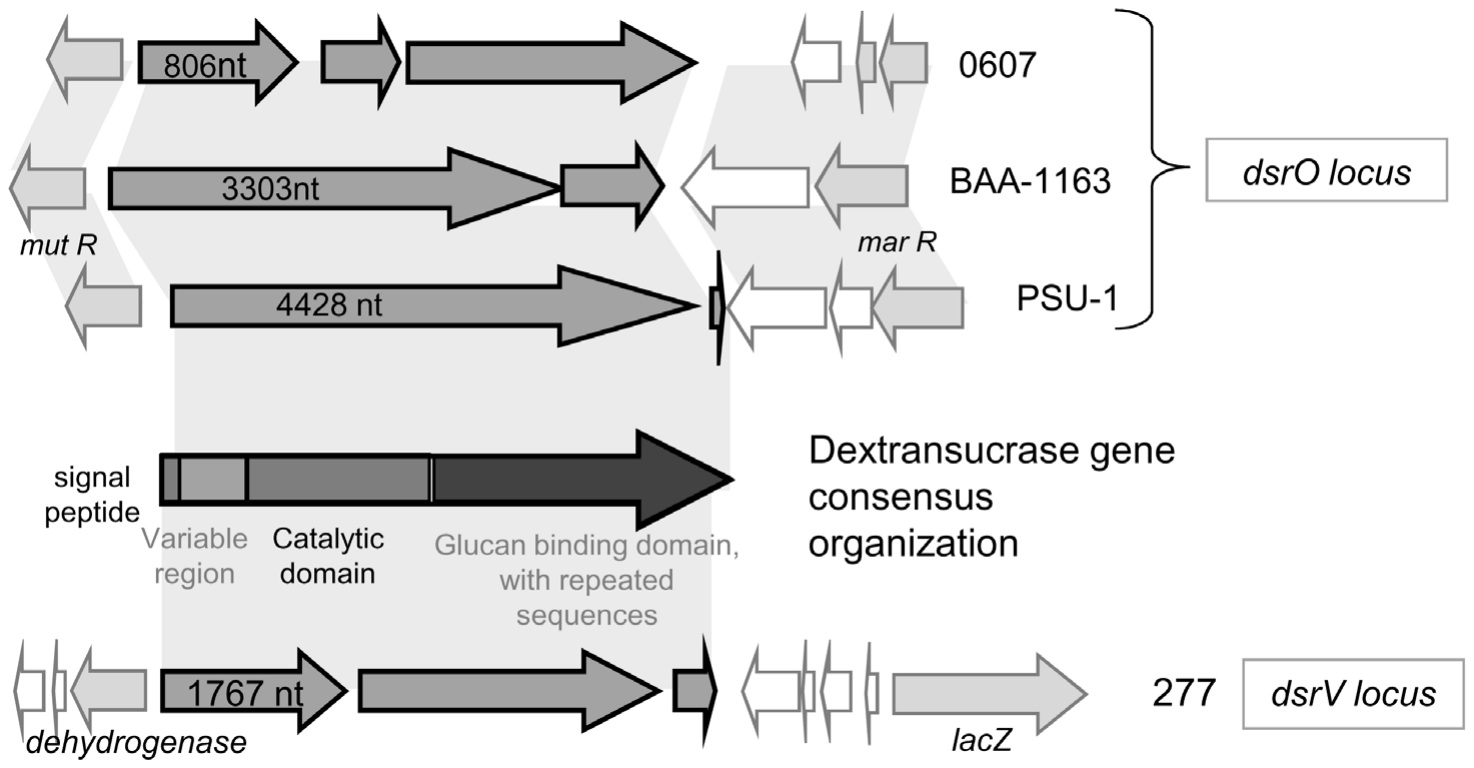
Genetic organization of *O oeni* chromosome regions harboring *dsrO* and *dsrV* genes. Example of strains *O. oeni* PSU-1, BAA-1163, 0607 and 277. The strain 277 also diplays a *dsrO* gene, similar to that found in *O. oeni* PSU-1.

Three glycoside hydrolases genes were identified. The first one, *dsrO,* was present in 49 genomes and always inserted in the same site on the chromosome (Figure 1). The entire sequence of this gene extended to 4428 nt (Figure 5). Point mutations could however shorten it, and modify the activity of the proteins produced. For example, for 10 out of 50 strains, *dsrO* had a stop codon at position 3303 nt, still generating a potentially active protein –as codons for amino acids of the catalytic triad were conserved [33]–[34]. For 4 strains out of 50, two stop codons in the sequence produced three ORFs, probably encoding inactive DsrO protein fragments. The protein DsrO was more than 90% conserved in the area preceding the mutation. In its long form (1475aa), it displayed 72% identity with the dextransucrase DsrP produced by *Leuconostoc mesenteroides* IBT-PQ (NCBI AAS79426.1) [35].

Eleven out of 50 genomes displayed an additional dextransucrase pseudogene (*dsrV*), whose sequence was 90% identical (100% coverage) between the genomes displaying it. However, the deduced protein was always truncated in the catalytic site, and may therefore be inactive in all cases (Figure 5). The position of the truncation varied depending on the strain studied. The identity between the genes *dsrO* and *dsrV* was 50%.

Thirteen out of 50 genomes had a levansucrase gene (*levO*), whose sequence was 98% identical between the strains displaying it. In strains 9304, C28 and S13, *levO* was cut prematurely, and most likely encoded an inactive enzyme. LevO displayed 49% identity with the putative levansucrase identified in *Oenococcus kitaharae* DSM17330 (WP_007744218.1), and 36% identity with the levansucrase LevS, produced by *Leuconostoc mesenteroides* B-512 F, characterized in 2006 [36].

Although present in a small number of genomes, and *levO* and *dsrV* genes were always inserted at the same site on the chromosome (Figure 1). Analysis of adjacent genes indicated the acquisition of *dsrV* could be linked to a phage attack (remnant) and rearrangements due to transposases. Regarding *levO*, no trace of mobile element nearby could explain the mode of acquisition of the gene.

### Distribution of *eps* Genes and phylogenetic tree

**Figure 6:**
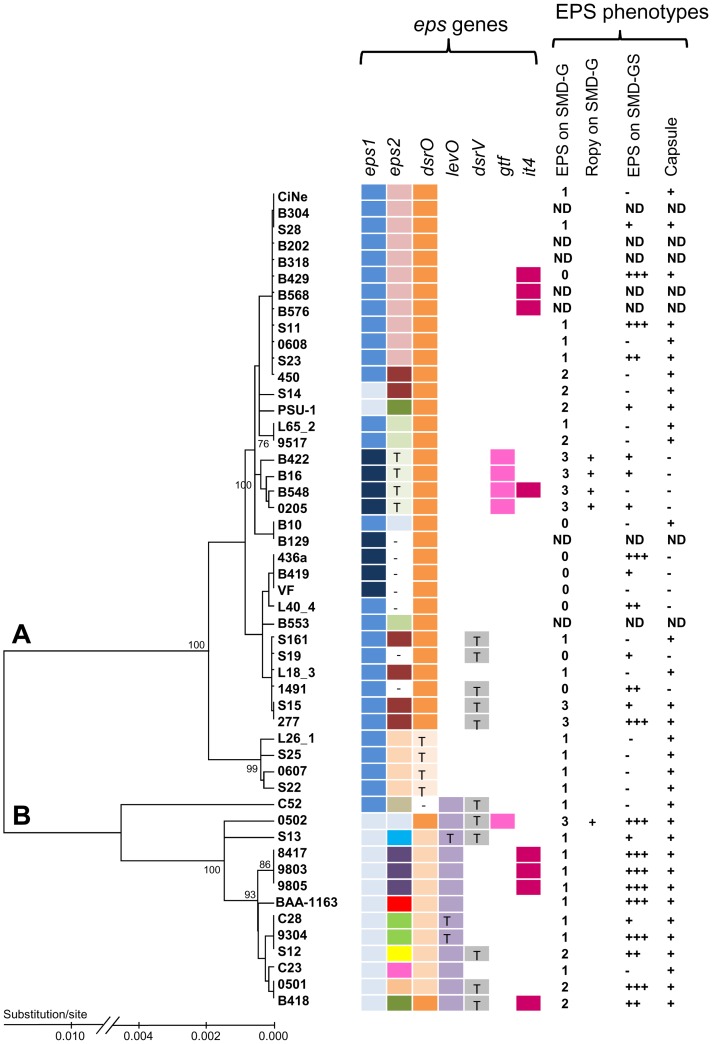
Distribution of *eps* genes and EPS phenotypes in the 50 *O. oeni* strains. The genome sequences were used for MLST typing in order to construct a consensus dendrogram, using the neighbor-joining method with bootstrap values (cut-off>70%). The two phylogroups A and B are indicated. Legend: ***eps1*** model: A: light blue, B: medium blue; C: dark blue; ***eps2***: each of the 14 complex models displays its own color, while the absence of *eps2* is indicated by a white box bearing the sign - and the presence of a truncated inactive *eps2* model is indicated by T. ***dsrO*** size: dark box: 4428 nt, medium color box: 3303 nt, light color box: 806 nt and white (−) box: no *dsrO*. ***levO*** is present when the box is parm and the symbol T indicates a truncated gene; *dsrV* is present when the box is gray and the symbol T indicates a truncated gene; ***gtf*** is present when the box is pink and ***it4*** is present when the box is garnet colored. For EPS production from glucose: 1: [EPS]<20 mg/l: 2: [EPS]<50 mg/l and 3: [EPS]>80 mg/l. The ropy phenotype is indicated by +. For EPS production from sucrose: +++: [EPS]>1000 mg/l: ++: [EPS]>250 mg/l and+[EPS]>100 mg/l. A white box (−) indicates an [EPS]<100 mg/l in the conditions of the assays. The incapacity to produce EPS from sucrose cannot be proved by this method. The presence of a capsule around the cells (negative staining) is indicated by+and its absence by -; Nd: not determined.

The 50 genome sequences were used for MLST typing using 6 housekeeping genes in order to construct a consensus dendrogram. The strains distributed into two main phylogroups (A and B), as previously described [11], [19]–[20]. The repartition of the *eps* genes and EPS phenotype on this dendrogram was then examined (Figure 6). All genomes in the branch B, except C52, displayed a model A of cluster *eps1*, while genomes in the branch A displayed the three models of cluster *eps1* (A, B or C). The strains having *levO* or the same version of *dsrO* were grouped on the phylogenetic tree. In contrast, the strains carrying *gtf*, *dsrV* or *it4*, putatively acquired via phage attack, were not grouped.

Regarding cluster *eps2*, strains that carried the same *eps2* model were generally grouped on the tree. For example, the 11 strains having a B429 model were all on the same branch. In other cases, strains with the same *eps2* are far apart on the tree: for example, strains displaying model PSU-1 or 0502 of *eps2* could belong to the A or B branches of the tree. In addition, strains belonging to remote subdivisions in branch A displayed the model 277 of *eps2* (450, S14, S161, L18_3, S15 and 277). In these cases, the acquisition of the *eps2* cluster may result from distinct events in the strains considered.

Some links between the *eps loci* appeared on the dendrogram. Actually, although strains with *eps2* model 277 or model 0501 sometimes have a model A of cluster *eps1* (450 or 0501), sometimes a model B of cluster *eps1* (277, S15, S161, L18_3 and B10), most of the time, when two genomes displayed the same cluster *eps2,* they also had the same *eps1*. Indeed, all the genomes with a cluster *eps2* model B429 or 0607 displayed a model B of cluster *eps1*, and all the genomes with a cluster *eps2* model 9805 or PSU-1 displayed a model A of cluster *eps1,* even if they are far apart on the phylogenetic tree. Furthermore, genomes with model C of cluster *eps1* systematically had a truncated or absent cluster *eps2.* In addition, genomes B422, B548, B16 and 0205, in which *eps2* cluster was strongly truncated (5.4 kb), were also those whose *gtf* gene was located in a phage remnant. The four strains, all from Champagne region [20], were grouped on the dendrogram. They may have diverged after the acquisition of their *eps* genes. In addition, in these 4 genomes, *gtf* may be “stabilized” compared to the genome 0502 which displayed *gtf* in a prophage and also a non truncated *eps2* cluster.

### Links between *eps* Genes and EPS Phenotypes

*O. oeni* is not amenable to genetic transformation. The consequence is that evidence for phenotype cannot be obtained by gene inactivation. As a result, we analyzed the phenotypes of a high number of strains, in order to identify potent links with the identified genotypes. Previous work suggested that, during growth in the presence of glucose as the sole carbon substrate, the EPS synthetic routes using nucleotide sugars were the sole active (Wzy dependent pathway and Gtf synthase pathway), whereas, in the presence of sucrose, the action of glycoside-hydrolases supplement the bacterial biosynthetic capabilities [16]. Phenotypes were therefore studied in the presence of glucose alone or in the presence of glucose and sucrose, most of the *O. oeni* strains studied being unable to use sucrose as a growth substrate [37]–[38].

In glucose-only medium, the strains studied produced low amounts of soluble EPS (<80 mg/l) with the exception of strains S15, 277 and of the 5 strains carrying the *gtf* gene (B422, B548, B16, 0205, and 0502), for which the medium also became ropy (Figure 6). The strain IOEB0205 is already known to produce β-glucan [14]. The 4 other ropy strains agglutinated in the presence of antibody targeting the β-glucan (not shown) indicating that they also produced this specific polymer. Except for these ropy strains, it was difficult to establish a link between the concentration of soluble EPS observed after growth in SMD-Glucose and the *eps* gene variants (Figure 6).

The monomer composition of the few soluble EPS produced on SMD-Glucose was investigated for a selection of 10 strains. All the genomes of the strains studied displayed *eps1* and *eps2* clusters. The strains 9803, 9805, PSU-1 9304 and S13 displayed a model A of *eps1*, while the others strains examined displayed a model B. Regarding *eps2*, the strains S11 and B429 had the same genotype (model B-429), the strains 9803 and 9805 had the same genotype (model 9805), and the others ones (9304–model 9304-, S13–model S13-, S22–model 0607-, PSU-1–model PSU-1-, 9517-model B553- and 277–model 277-) displayed different genotypes (figure 6). Soluble polysaccharides obtained after growth in SMD-glucose medium were of moderate size (less than 400 kDa). Whatever the strain studied, the soluble EPS produced on SMD-glucose medium only contained glucose, galactose and rhamnose. No trace of osamine, pyruvate, acetate, glycerol or uronic acid was detected.

**Figure 7:**
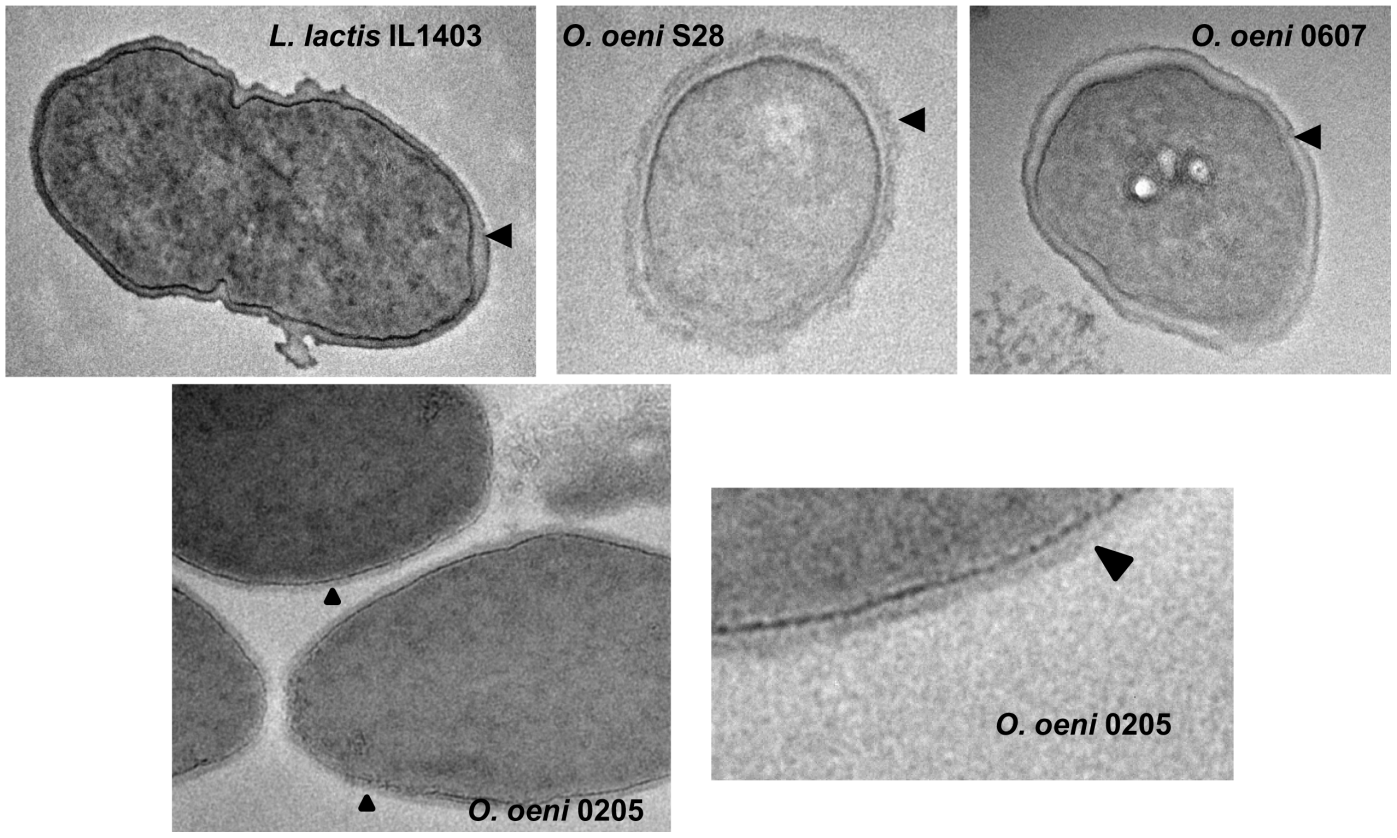
Observation of *O. oeni* capsules by transmission electron microscopy. The black arrow indicates the place where the capsule may appear as a dark halo/layer when present. The strain *L. lactis* IL1403, which displays a thin polysaccharide pellicle as demonstrated by Chapot Chartier et al. [70], serves as a reference. Strains *O. oeni* S28 and 0607 are clearly encapsulated, while strain 0205 has no dense area beyond the peptidoglycan layer (light gray layer).

The low level of EPS production on SMD-glucose prompted us to look for the presence of capsular polysaccharides. Indeed, after growth on either SMD-glucose or grape juice medium, most of the studied bacteria appeared encapsulated (Figure 6). Only the bacteria having a highly truncated or no *eps2* cluster showed no capsule, whatever the model of cluster *eps1* they displayed: model B (1491 or L40_4) or model C (B129, 436a, B419, VF, B422, B16, B548 or 0205). Observed by transmission electron microscopy, this capsule was thicker or thinner depending on the strain (Figure 7). Monomer composition analysis of the capsular EPS of strains 9304, S28 and S11 gave the following results : 9304 (Galactose : Glucose : Rhamnose, 68.4∶ 15.2∶ 6.9), S28 (Galactose : Glucose : Rhamnose, 41.7∶ 35.2∶ 11.1) and S11 (Galactose : Glucose : Rhamnose, 41.2∶ 31.2∶ 20.7). The strains S28 and S11, which displayed the same *eps* genotype, produced capsular polymers with close monomer composition compared to strain 9304 which displayed a different *eps* genotype.

The addition of sucrose to the medium induced a marked overproduction of exopolysaccharides with some strains (Figure 6), although 75% did not use sucrose as a growth substrate. The EPS produced in the presence of sucrose being considerably more abundant, more precise structure analyses could be made (Table 3). First, analysis of the culture supernatants by size exclusion chromatography indicated that the addition of sucrose to the culture medium induced the appearance of a peak corresponding to additional polymers of very high molecular weight (6 000 to 10 000 kDa), with all the strains examined, except strain S25. This last strain was the only one in Table 3 which did not encode a functional glycoside-hydrolase. The structure of the high molecular weight polymer was determined. In all cases, the peak contained a homopolysaccharide or a homopolysaccharide mixture. All strains having a functional *dsrO* gene (gene length ≥ 3303 nt) produced a 1,6 linked glucan displaying about 5% 1,3 branches. Hydrolysis of the polymer by dextranase confirmed this was an α-glucan (dextran). Besides dextran, strains BAA- 1163 and 0501 produced a 2.6-bound fructan. This fructan contained links with β configuration (Vuillemin, unpublished data).

**Table 3:** Structural analysis of the soluble exopolysaccharides produced by selected strains.

Strain^a^	Glycoside-hydrolase genes*	EPS produced on SMD-glucose^b^ mg.l^−1^	EPS produced on SMD-glucose-Sucrose^b^ mg.l^−1^	Structure of the EPS of high molecular weight (>30 kDa) produced on SMD-Glucose-sucrose^c^
S25	*dsrO* (806 nt)	20±3 (1)	85±4 (1)	ND
9517	*dsro* (4428 nt)	76±6 (1)	95±6 (2)	Glucan 92% (no linkage determination)
S11	*dsrO*(4428 nt)	38±5 (1)	3867±23 (2)	100% glucan (95% 1,6 linked, 5% 1,3 linked)
B-429	*dsrO* (4428 nt)	10±3 (1)	2080±125 (2)	100% glucan (95% 1,6 linked, 5% 1,3 linked)
9304	*dsrO* (3303 nt) *levO* (truncated)	37±6 (1)	1848±119 (2)	100% glucan (93% 1,6 linked, 7% 1,3 linked)
BAA-1163	*dsrO* (3303 nt) *levO*	31±5 (1)	1819±157 (2)	40% glucan (95% 1,6 linked, 5% 1,3 linked) and 60% fructan (2,6 linked)
0501	*dsrO* (3303 nt) *levO, dsrV truncated)*	55±6 (1)	3288±210 (2)	5% Glucan and 95% fructan (2,6 linked)

a All the strains in the Table also displayed *eps1* and *eps2* clusters. None displayed *gtf.*

b The EPS concentration was determined by the anthrone sulfuric method. The number between brackets indicates the number of chromatographic peaks after gel permeation on superdex 30 column. The peak at 5500 Da was always present. The second peak, when present indicates the presence of polymers with molecular weight higher than 1 000 000 Da.

c ND: not determined, no high molecular weight EPS produced.

The strains which were not able to produce EPS from sucrose displayed different glycoside-hydrolase genotype and links between genotype and phenotype were not obvious. Indeed, the lack of EPS synthesis from sucrose is coherent in the case of strains with only a truncated dextransucrase *dsrO* (strains 0607, S22, S25, L26_1). However, it cannot be explained, for many others, by the absence or mutation of glycoside-hydrolase genes (i.e. in some strains with a *dsrO* gene 3303 to 4428 nt long, such as CiNe, 0608, S14 and many others, Figure 6).

## Discussion

*Oenococcus oeni,* which drives malolactic fermentation in most wines (especially red ones) and ciders, is very rarely encountered elsewhere or at other stages of winemaking. This is a unique and perfectly specialized bacteria [9]. The analysis of 50 genomes of *O. oeni* shows that genes dedicated to EPS metabolism are distributed all around the chromosome. The *eps loci* are numerous (*eps1*, *eps2*, *dsrO*, *dsrV*, *levO*, *gtf*, *it3*, *it4*) and often divergent from one genome to another. This high diversity fully justifies the method chosen to establish an inventory of *eps* genes (genome sequencing). Genes of interest were identified on the basis of sequence homology, as proposed in other studies [39]. Though the matrix genes blasted in our study are much more numerous (82 reference genes instead of one single gene of priming glycosyltransferase), the existence of genetic determinants with widely differing sequence cannot completely be excluded. However, we found a large number of genes potentially involved in the production of EPS, whose presence is generally relatively well correlated with the observed phenotypes. This suggests that the majority of genes of interest were identified. It appeared that the strains that induced medium ropiness all display *gtf* and produce β-glucan. They represent 10% of the strains in the collection studied, while previous work reported a 22% prevalence for *gtf* [14]. The strains that produce β-fructan in the presence of sucrose all exhibit a non truncated levansucrase gene, *levO*. The prevalence of *levO* is 26%, with levan production in 77% of the *levO* strains. Regarding dextran synthesis and dextransucrase gene (*dsrO*), the relationship between genotype and phenotype is less clear. Indeed, the presence of functional genes is not always sufficient to explain the observed phenotypes. Gene expression and activity of DsrO could be modulated by certain environmental factors or the physiological state of cells. In previous studies, we observed that glucan and fructan production from sucrose was not detectable in MRS medium but only in semi defined one [15]–[16]. Anyway, the glycoside-hydrolases of *O. oeni* are not original as regards both the protein primary structure and the structure of the polymers produced. All the encapsulated *O. oeni* strains displayed a cluster *eps2* which encodes the proteins necessary for reconstituting a wzy-dependent pathway. The absence or the significant truncation of cluster *eps2* are always associated with the absence of the polysaccharidic capsule. Nevertheless, the fact that the strain BAA-1163 is encapsulated, although its *eps2* cluster lacks the priming glycosyltransferase, suggests that internal complementation for priming glycosyltransferase is possible (for example by means of genes *woaA* or *it3*). In all cases examined, the capsular polymer contains glucose, galactose and rhamnose. This close monomer composition contrasts with the vast diversity of *eps2* cluster sequences. Differences in the osidic bounds encountered in the repeating unit could still exist, and further structure analyses will be necessary to establish a link between the transferases and the monomers present.

The role of cluster *eps1* and of the isolated genes *it3* and *it4* could not be determined in this study. The advantage of the presence of two *eps* clusters remains obscure, but it is clear that this is a common feature to all genomes in the species. Moreover, this is also the case for *O. kitaharae*, the other species in the genus *Oenococcus* [40]. Analysis of conserved domains did not enable to clearly predict the function of the Wzy protein encoded in *eps1* (polymerase or ligase). If Wzy is a polymerase, then *eps1* operon would direct the synthesis of an exopolysaccharide. The wzy-dependent synthesis route would be duplicated (one being encoded by *eps1* and the other by *eps2)* with production of two distinct polysaccharide structures, as described for other lactic acid bacteria [41]–[42]. On the other hand, if the wzy gene in *eps1* encodes a ligase (WaaL), the cluster *eps1* may direct the synthesis of an oligosaccharide wherein the ligase then fixes a polysaccharide synthesized by proteins encoded in another cluster (*eps2* for example), on the model of lipopolysaccharide of Gram-negative bacteria [43]–[44]. In both cases, the product whose synthesis is directed by the *eps1* should be minor because (i) glucuronic acid and phosphoglycerol are never found in the structural analysis of the EPS examined (either soluble or capsular), and (ii) the strains lacking *eps2* cluster but displaying *eps1* show no capsule and produce very low level of soluble EPS in SMD-Glucose.

The distribution of the *eps* genes on the phylogenetic tree is complex. Some genes have clearly been acquired by horizontal transfer after the attack of a bacteriophage (*it4*, *gtf*, *dsrV*), while others, could have been acquired earlier in the history of the species (*levO*, *dsrO*, *eps1*) or could result of very numerous chromosome modifications (*eps2*). The *eps2* clusters are the most polymorphic among the studied *loci*. Such a diversity (15 cluster models for 50 genomes) is surprising in a non-pathogenic bacterium as it resembles what is described in *Streptococcus pneumoniae*, in which, *eps* clusters direct the synthesis of a major virulence factor, the pneumococcal capsule [45]. Regarding the cluster organization, the *eps2* clusters, inserted between *amiO* and *recP* also strongly resemble those described for streptococci, whether *S. thermophilus*, in which the *eps loci* are inserted between genes *deoD* and *pgm*, or *S. pneumoniae,* in which *cps loci* are inserted between genes *dexB* and *aliA* [46]–[47] or for *Lactococci* or *Lactobacilli* [48]–[50]. Genes *dexB* and *aliA* are spaced by 10 to 30 kb maximum [47], while *amiO* and *recP* and genes can be distant from 50 kb. This region is the most heterogeneous in the *O. oeni* chromosome [51]. According to Golubchik et al. [52], the acquisition of *eps* cluster may be accompanied by a large number of changes, spread all along the chromosome. The acquisition of the *eps2* could thus be the cause of the divergence of certain genomes. Loss of cluster *eps2* is rare and in some cases, it is accompanied by the acquisition of the *gtf* gene (Champagne strains). The presence of a truncated *eps2* could have been a selection pressure for the stabilization of *gtf* (phage remnant). This situation reminds again, what is described in *S. pneumoniae* Type 37 [53].

The fact that the 50 genomes studied possess genes dedicated to EPS metabolism suggests that these polymers are very important for the adaptation of *O. oeni* to its ecological niche. This is even more true for *eps* clusters, not only because they occupy a significant portion of the *O. oeni* small chromosome, but also because the biosynthetic pathway encoded (wzy dependent) is energy consuming [9], [54]–[56]. It is generally claimed that capsular polysaccharides have a mainly protective role while free EPS are interesting from a technological point of view [49], [57]. The production of soluble polysaccharides by the strains studied is low in the absence of sucrose (<80 mg/L), but similar to that described for some other lactic acid bacteria [14], [16], [49], [55]–[56], or for *O. oeni* in wine [13]. Thirty-two out of 43 strains examined are encapsulated (75%), against 30% for *S. thermophilus* [57] or 50% for *S. pneumoniae* [47]. In *S. pneumoniae*, the capsule is an essential virulence factor. The capsule could thus be a key element for *O. oeni* survival in a hostile environment. In general, capsular EPS do not constitute an energy supply for the cell that produces them [58]–[59]. These should rather constitute a protective layer against desiccation, osmotic acid or cold stress, digestion by lysozyme, or against toxic compounds such as alcohol or sulphur dioxide [50], [60]–[63]. EPS could also play a role in biofilm formation, thereby facilitating the colonization of various ecosystems and especially grapes pellicules, barrels and other wine-making material [14], [44], [59], [64]–[66]. As regards the protection against phage attacks, opposite effects have been described: certain EPS are specifically recognized by certain phages and predispose bacteria to the attack by these phages, while others would be a protective barrier [57], [67]. It might be interesting in the future to connect the diversification of *eps* genes with the high variability in Oenophages recently described [12], [68], [69].

## Supporting Information

Table S1 ***In silico*** **inventory of** ***eps*** **genes.** List of *eps* genes encountered in the initial database and then, in the 50 genome sequences studied, *locus* by *locus* (*eps1* and *eps2* clusters, isolated glycosyltransferase and glycoside hydrolase genes, and genes involved in precursor synthesis).
(XLSX)
